# Impact of the Expert Consensus on Carbapenem Consumption Trends and Patterns in Public Healthcare Institutes: An Interrupted Time Series Analysis, 2017–2020

**DOI:** 10.3389/fphar.2021.739960

**Published:** 2022-01-13

**Authors:** Dan Ye, Caijun Yang, Wenjing Ji, Jie Zheng, Jingyi Zhang, Runqing Xue, Jianli Gu, Minchun Chen, Kangkang Yan, Yongzhong Liu

**Affiliations:** ^1^ Department of Pharmacy, Xi’an No.3 Hospital, The Affiliated Hospital of Northwest University, Xi’an, China; ^2^ Xi’an Key Laboratory of Cardiovascular and Cerebrovascular Diseases, Xi’an No.3 Hospital, The Affiliated Hospital of Northwest University, Northwest University, Xi’an, China; ^3^ Department of Pharmacy Administration and Clinical Pharmacy, School of Pharmacy, Xi’an Jiaotong University, Xi’an, China

**Keywords:** antibacterial consumption, carbapenem, interrupted time series, expert consensus, China

## Abstract

**Background:** Carbapenems are considered the last line of defence against bacterial infections, but their high consumption and the resulting antibacterial resistance are an increasing global concern. In this context, the Chinese health authority issued an expert consensus on the clinical applications of carbapenems. However, the long- and short-term effects of the expert consensus on carbapenem use are not clear.

**Methods:** This study was conducted in Shaanxi, a northwest province of China. We collected all available carbapenem procurement data between January 2017 and December 2020 from the Provincial Drug Centralized Bidding Procurement System. A quasi-experimental interrupted time series analysis was used to evaluate the longitudinal effectiveness of expert consensus by measuring the change in the Defined Daily Dosesper 1,000 inhabitants per day (DID), the percentage of carbapenem expenditures to total antimicrobial expenditure, the total carbapenem expenditure, and the defined daily cost (DDDc). We used Stata SE version 15.0 for data analysis, and *p* < 0.05 was considered statistically significant.

**Results:** After the distribution of the expert consensus, the level (*p* = 0.769) and trend (*p* = 0.184) of DID decreased, but the differences were not statistically significant. The percentage of carbapenem expenditures to total antimicrobial expenditure decreased abruptly (*p* < 0.001) after the intervention, but the long-term trend was still upward. There was no statistically significant relationship between the release of the expert consensus and carbapenem expenditure in the long term, but there was a decreasing trend (*p* = 0.032). However, the expert consensus had a positive impact on the economic burden of carbapenem usage in patients, as the level (*p* < 0.001), and trend (*p* = 0.003) of DDDc significantly decreased.

**Conclusion:** The long-term effects of the distribution of the expert consensus on the use and expenditure of carbapenems in public health institutions in Shaanxi Province were not optimal. It is time to set up more administrative measures and scientific supervision to establish a specific index to limit the application of carbapenems.

## Introduction

Globally, the inappropriate use of antibacterial agents and the resulting bacterial resistance has created a serious threat to public health, economic growth, and global economic stability (Watson, 2017; Kuehn, 2018). There are more than 2.8 million drug-resistant infections reported each year in the United States, resulting in more than 35,000 deaths (Centers for Disease Control and Prevention, 2019). In the absence of an effective strategy to control antibacterial resistance, it is estimated that by 2050, there will be more than 10 million deaths each year globally caused by resistant infections (O’Neill, 2016). In such a setting, common treatments such as general surgery and chemotherapy could not be carried out due to bacterial resistance. In China, the morbidity and mortality of diseases caused by multidrug-resistant bacteria and pandrug-resistant bacteria are higher than those in other countries (Jiancong et al., 2020). The 2020 China Antimicrobial Surveillance Network (CHINET) reported that the proportions of *Escherichia coli* resistant to third-generation cephalosporins, carbapenem-resistant *Klebsiella pneumoniae* (KPN) and *Acinetobacterbaumannii* (AB) were 55.5, 23.7 and 73.2%, respectively (China Antimicrobial Surveillance Network, CHINET, 2020).

To combat bacterial resistance, the National Health and Family Planning Commission of the People’s Republic of China has issued many regulations for the clinical use of antibacterial agents. With the establishment of a relatively complete antibactical drug management system and technical support framework, antibactical resistance monitoring and antibactical drug use monitoring networks, the use of antimicrobial agents in healthcare institutions has been greatly improved in mainland China (Xiao, 2018). However, the irrational use of antibacterial agents is still obvious in clinical practice. A large-scale study investigated the appropriateness of antibiotic prescriptions in ambulatory care settings which conducted in mainland China, and found that 51.4% of antibiotic prescriptions were inappropriate and 8.4% were potentially appropriate, significantly higher than those in Europe and the United States (Zhao et al., 2021).

Carbapenems as a class of antibiotics with broad-spectrum, strong antibacterial activity and high stability to β-lactamase, are considered Watch antibiotics according to the AWaRe classification framework of WHO (WHO, 2019). In mainland of China, carbapenems are considered the last line of defense against bacterial infections due to their higher antimicrobial resistance potential. Many studies have shown that carbapenem consumption may be a risk factor for subsequent infection with carbapenem-resistant Gram-negative bacteria (Hu et al., 2016; Liu et al., 2018), bringing great challenges to the treatment of critically ill patients. During the past 15 years, the prevalence of meropenem-resistant KPN increased from 2.9 to 24.2%, and that of imipenem-resistant AB increased from 32.9 to 72.9% (China Antimicrobial Surveillance Network, CHINET, 2020). The phenomenon of irrational use of carbapenems should be given more attention in China.

In response to the appreciably increasing in carbapenem-resistant bacteria, the health authority issued a notice on “the issuance of three technical documents concerning expert consensus on the clinical application of carbapenems” (hereafter referred to as the expert consensus), which put forward corresponding regulations for the clinical indications of carbapenems and their applications for special populations (National Health and Family Planning Commission of Shaanxi Province, 2018b). The content of expert consensus provides detailed explanations on the clinical indications, variety selection, dosing regimens, etiology and efficacy evaluation, prescription authority of carbapenems, and also the evaluation indicators for the rationality of clinical application of carbapenems antibiotics. The higher-level competent authority issued the document to each public medical institution, and informed the medical institution to learn the content of the document, and ensure the use of carbapenem in accordance with the guidance given in the document. At the same time, medical institutions are required to register each patient’s situation of carbapenem used in accordance with the evaluation rules to achieve special management.

However, the long- and short-term effects of the expert consensus on carbapenem use are not clear. The main aim of this study was to determine the trends in carbapenem consumption at public healthcare institutions from 2017 to 2020. The secondary aim was to evaluate the impact of the promulgation of the expert consensus on the use of carbapenems, providing an empirical reference for the formulation of antibacterial management policies.

## Methods

### Study Setting

Northwest China, where Shaanxi Province is an important hub of the One Belt and One Road national strategy and a major pilot province for Western health system reform, had a population of 39.52 million, and an area of 205,800 square kilometres in 2020. The annual disposable income of Shaanxi in 2019 was $3,783.17 (based on the value of US$1 = 6.52 RMB in 2020), ranking 19th among all 31 provinces and first in northwestern China. In 2019, Shaanxi had 2.80 (2.05–3.84) physicians, 3.88 (2.57–5.37) nurses, and 6.86 (6.30–8.44) beds per 1,000 residents in the health care sector. The health authority of Shaanxi Province issued the expert consensus on October 30, 2018, requiring further strengthening of the monitoring and evaluation of the use of carbapenems (National Health and Family Planning Commission of China, 2018a).

### Data Collection

In this study, data on the use of carbapenem agents from 2017–2020 were obtained from procurement records from the Shaanxi centralized bidding procurement system. More than 95% of pharmaceuticals used by public healthcare institutes must be purchased through this system (Shaanxi Provincial Department of Health, 2012). In 2020, a total of 2,292 public healthcare institutes procured drugs through this province-wide platform, including 256urban medical institutions, 290 county-level medical institutions, and 1,746 primary medical institutions (Table 1).

**TABLE 1:** Number of public healthcare institutions in Shaanxi Province, 2017–2020.

Year	Urban hospitals	County-level hospitals	Primary healthcare institutes	Total
2017	241	277	1726	2,244
2018	223	280	1711	2,214
2019	238	287	1733	2,258
2020	256	290	1746	2,292

### Data Analysis

A quasi-experimental interrupted time series (ITS) analysis was used to evaluate the longitudinal effects of the expert consensus. ITS could profoundly reflect the impact of the intervention on the outcome, including the degree of improvement, whether it is immediate or delayed, and whether it is transient or long-term (Wagner et al., 2010). The intervention time in our study began in November 2018, as the expert consensus was issued by the Shaanxi Provincial Health Authority on October 30, 2018. This study covered every month continuously for an overall period of 48 months, consisting of 22 months before the intervention and 26 months after the intervention. The Durbin-Watson statistic was used to test whether there was any autocorrelation, and the Dicky-Fuller statistic was used to examine seasonal fluctuations. We used Stata SE version 15.0 for data analysis, and *p* < 0.05 was considered statistically significant.

### Indicators

The consumption of carbapenem was the total of sales and expressed as the number of DDDs per 1,000 inhabitants per day (DIDs) (Klein et al., 2021). The number of inhabitants was counted based on the year-end population reported by the Statistical Year Reports of Shaanxi (The Statistical Yearbook in Shaanxi Province in 2019, 2019). According to WHO/ATC recommendations, Defined Daily Dose (DDD) is the average daily maintenance dose of medication for adults for primary therapeutic purposes (WHO, 2013). To make the procurement data comparable before and after the intervention, the DDD value of meropenem in this study was set as 3. DDDs is the total consumption of antibiotic agents divided by DDD, which was calculated as the number of unit strengths (g) × pack size × amount/DDD (g/IU).

The expenditure of carbapenem was assessed using the total monthly expenditure for carbapenem (US dollars) (Yin et al., 2018). The total expenditure was calculated by adding up the cost for each procurement, which was equal to the quantity purchased per purchase multiplied by the unit price. And other outcome measures were the percentage of carbapenem expenditure to total antimicrobial expenditure, and the defined daily cost (DDDc). DDDc represents the total price level of carbapenems, which was equal to the total expenditure divided by DDDs. The larger the DDDc, the heavier the economic burden on the patients.

## Results

**FIGURE 1:**
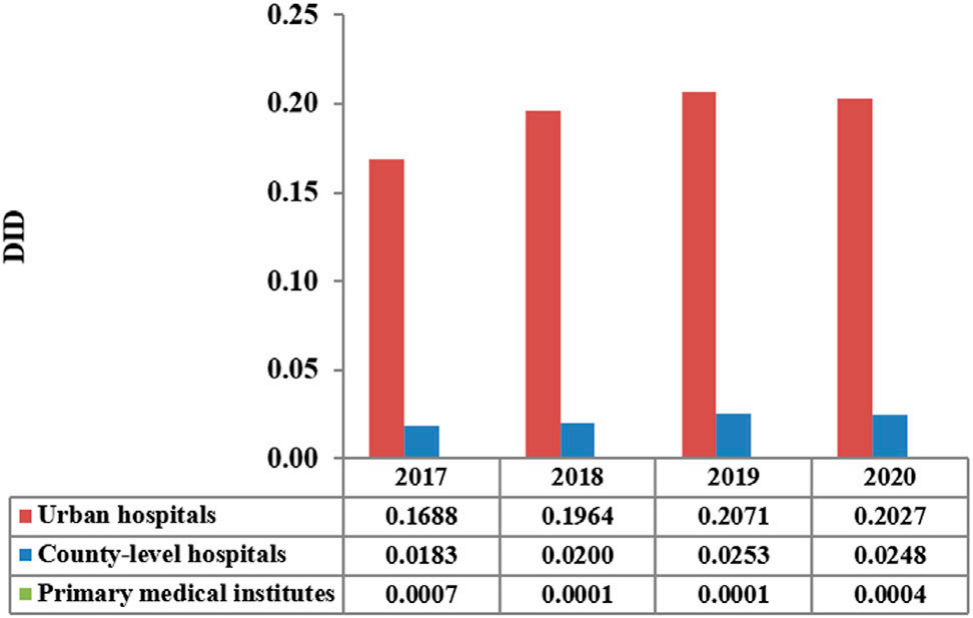
DID of carbapenems by the level of healthcare institution in Shaanxi Province, 2017–2020.

**FIGURE 2:**
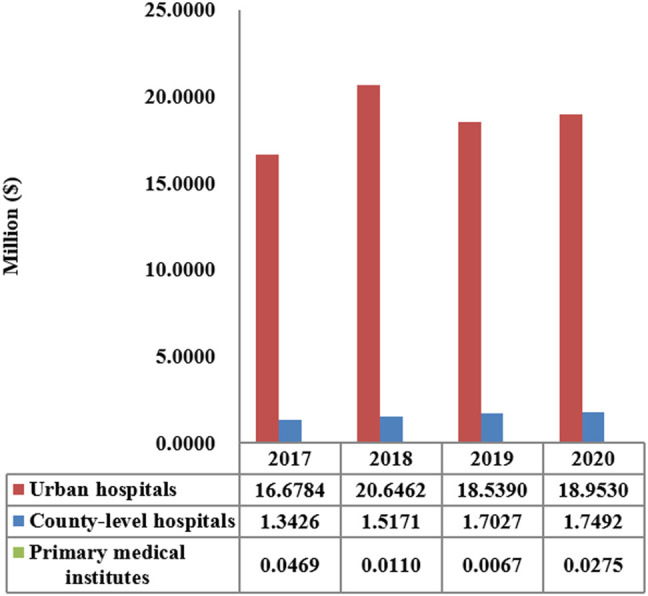
Total carbapenem expenditure by the level of healthcare institution in Shaanxi Province, 2017–2020.

**FIGURE 3:**
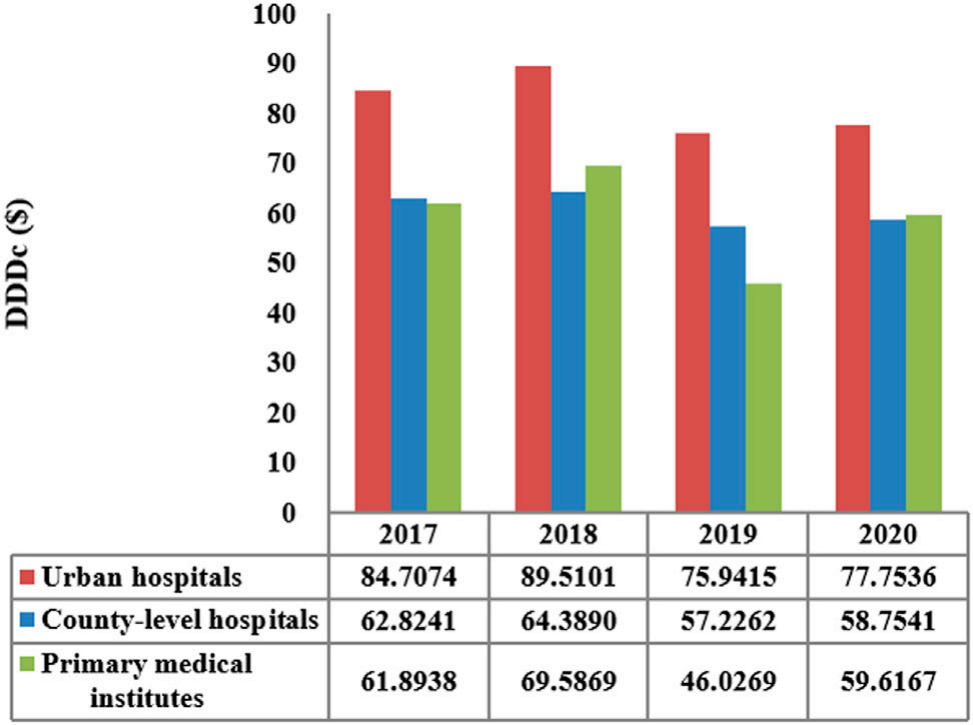
DDDc of carbapenems by the level of healthcare institution in Shaanxi Province, 2017–2020.

The total consumption of carbapenem agents in Shaanxi Province increased from 0.1878 DID in 2017 to a peak of 0.232 DID in 2019, and thereafter decreased to 0.228 DID in 2020. More than 85% of the carbapenems were used in urban hospitals (Figure 1). The overall expenditure on carbapenems among public healthcare institutes in 2020 was $20.73 million, 17% higher than that in 2017 ($18.07 million), and Figure 2 shows the specific expenditure on carbapenem agents at different levels of health institutes. Figure 3 shows that the burden of the carbapenem agents used by the patients decreased year by year, from 82.46$ in 2017 to 75.66$ in 2020, and it was higher in urban hospitals than in county-level hospitals and primary health institutes.

### The Effects on Carbapenem Consumption

**FIGURE 4:**
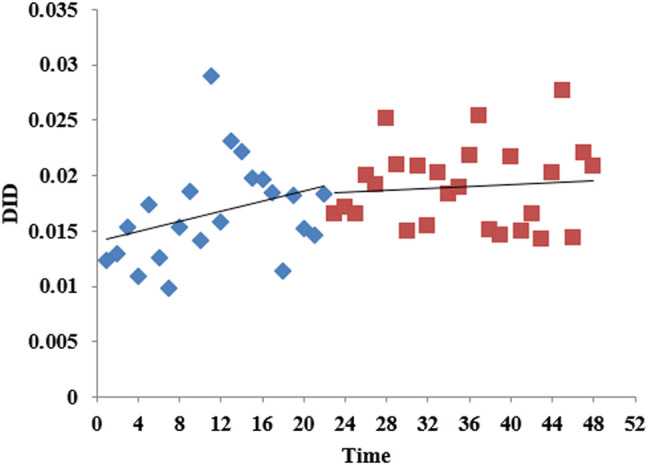
The consumption of carbapenems in Shaanxi Province, 2017–2020.

According to the time series of the consumption of carbapenems by month shown in Figure 4, DID fluctuated between 0.01 and 0.03 over time. Before the intervention, the consumption of carbapenems slowly increased, and after the intervention, the rate of increase slowed down. Table 2 shows that there was no obvious level (*p* = 0.769) or trend (*p* = 0.184) change before and after the release of the expert consensus. The analysis of different levels of healthcare institution showed that there was no statistical difference between the level and trend of carbapenem consumption in municipal and county hospitals and the promulgated of expert consensus. The trend of carbapenem consumption in primary medical institutions increased after the intervention, and the difference was statistically significant (*p* = 0.015), although the level (*p* = 0.904) of change was not significant. For details, see Supplementary Tables S2–S4.

**TABLE 2:** The impact of the implementation of the expert consensus on the use of carbapenems in Shaanxi.

Indicators	Coefficient	t	*p*-value	95% CI
DID				
β_0_	0.0139,874	9.99	0.000	0.0111656, 0.0168093
β_1_	0.0002286	2.25	0.029	0.0000241, 0.0004332
β_2_	−0.0005676	−0.30	0.769	−0.0044378, 0.0033026
β_3_	−0.0001878	−1.35	0.184	−0.0004678, 0.0000923
Percentage of carbapenem expenditure to total antimicrobial expenditure				
β_0_	0.0571519	11.38	0.000	0.0470318, 0.0672721
β_1_	0.0006358	1.90	0.064	−0.0000377, 0.0013093
β_2_	−0.0229872	−4.84	0.000	−0.0325688, 0.0134056
β_3_	−0.0001394	−0.36	0.721	−0.0009205, 0.0006417
Total expenditure				
β_0_	1268209	9.51	0.000	999523.6, 1536895
β_1_	33146.6	3.24	0.002	12524.54, 53768.67
β_2_	−346244.4	−1.98	0.054	−698974.2, 6485.401
β_3_	−28817.78	−2.22	0.032	−55013.77, 2621.794
DDDc				
β_0_	79.4137	50.02	0.000	76.2118, 82.6156
β_1_	0.5233181	4.80	0.000	0.3036175,0.7430187
β_2_	−12.8989	−4.01	0.000	−19.38485, 6.412949
β_3_	−0.6384013	−3.11	0.003	−1.052958, 0.2238442
season	−1.156,585	−0.99	0.328	−3.512294,1.199123

### The Effects on the Economic Burden of the Residents

**FIGURE 5:**
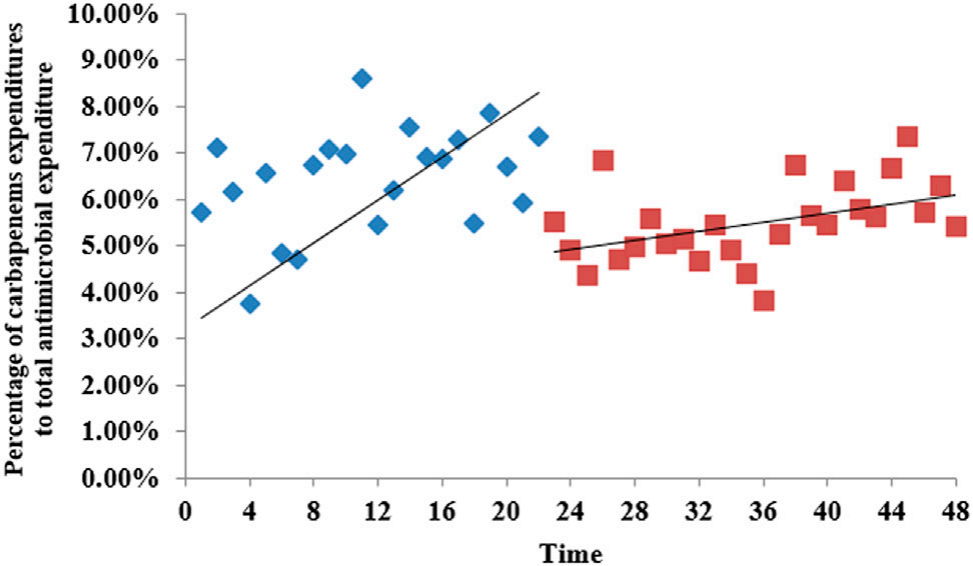
The percentage of carbapenem expenditure to total antimicrobial expenditure, 2017–2020.

According to the time series of the percentage of carbapenem expenditure to total antimicrobial expenditure in Shaanxi Province public hospitals in Figure 5, there was a slow increasing trend before the intervention, and after the intervention, the slope changed suddenly, but the trend was still upward. Table 2 shows that after the intervention, there was a significant decrease (*β*_*2*_ = −0.0229872, *p*<0.001) in the intercept. The level of it in urban medical institutions decreased significantly (*β*_*2*_ = −0.0187509, *p* = 0.002) after intervention (Supplementary Table S2). For primary medical institutions, the trend increased (*β*_*3*_ = 0.0001237, *p* = 0.012) after intervention (Supplementary Table S4).

**FIGURE 6:**
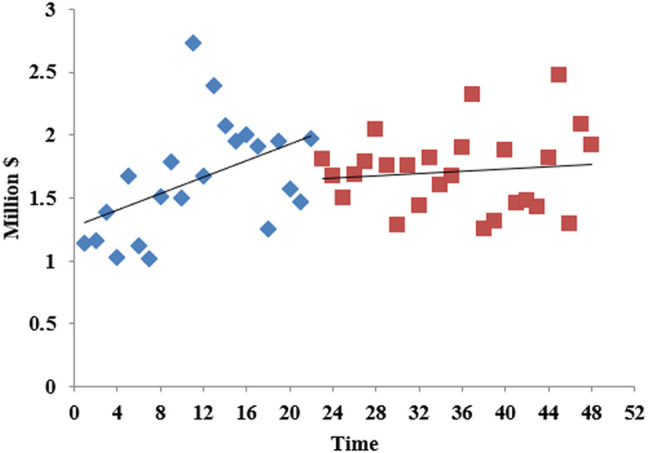
The total expenditure on carbapenem in Shaanxi Province, 2017–2020.

The overall carbapenem expenditure steadily increased between 2017 M1 and 2020 M12. Before the intervention, the increase rate was large; after the intervention, the horizontal intercept decreased slightly, and the trend change rate was small (Figure 6). As seen in Table 2, there was no statistically significant relationship between the introduction of the expert consensus and carbapenem expenditure in the long term, but there was a decreasing trend (*β*_*3*_ = −28817.78, *p* = 0.032). Supplementary Table S2 showed that the level (*β*_*2*_ = −2582,325, *p* = 0.031) and trend (*β*_*3*_ = −189127.8, *p* = 0.033) of carbapenem expenditure in urban hospitals significantly declined after the expert consensus was promulgated. For primary medical institutions, the trend increased (*β*_*3*_ = 0.0002673, *p* = 0.037) after intervention (Supplementary Table S4).

**FIGURE 7:**
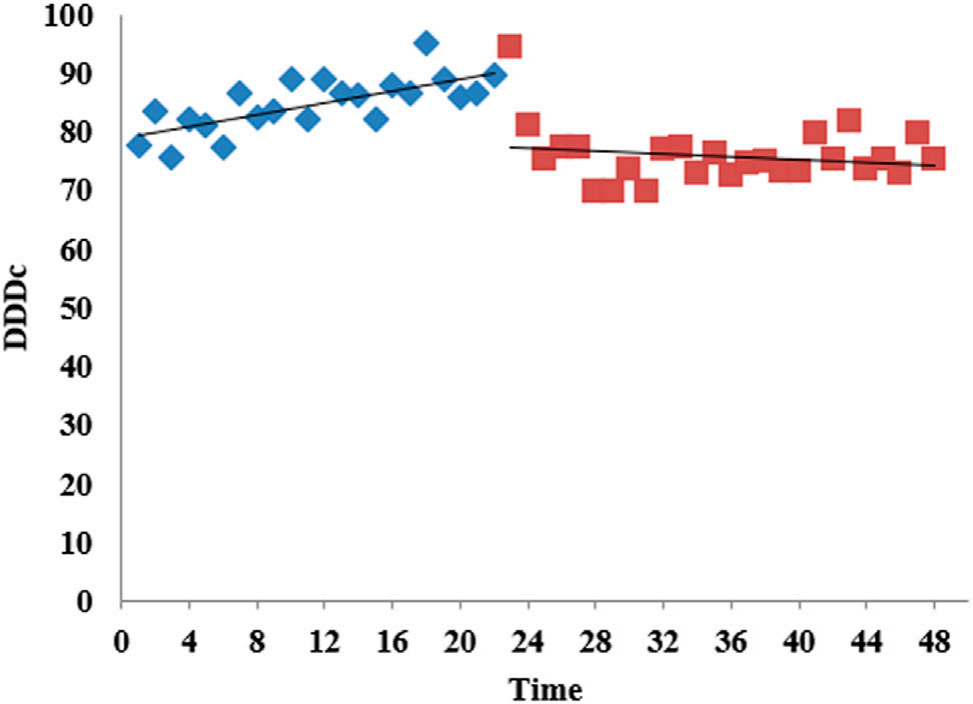
The economic burden of residents using carbapenem agents in Shaanxi Province, 2017–2020.

Figure 7 shows the change in carbapenem DDDc from M1 to 2020 M12 in 2017. It shows an upward trend month by month before the intervention and reaches a peak of 94.3923$ in 2018 M11, with a slow downward trend after the intervention. After the intervention, the level of DDDc decreased (*β*_*2*_ = −12.8989, *p*<0.001) and the trend also decreased (*β*_*3*_ = −0.6384, *p* = 0.003), and both were significant. The changes of urban hospitals are consistent with the overall situation (Supplementary Table S2).

## Discussion

To the best of our knowledge, this is the first quantitative survey using interrupted time series analysis to explore the impact of the issuance of the expert consensus on the long-term trend of carbapenem consumption in western China. Through time series analysis, we revealed the effect of this administrative regulatory measure on the usage of carbapenems, which was not examined in previous studies.

### The Effects on Carbapenem Consumption

This study found that the total consumption of antibiotics increased from 0.187 DID in 2017 to 0.228 DID in 2020, which was much higher than that in other developed countries, indicating that the overuse of carbapenems is a serious problem in China. A study conducted in South Korea found that the consumption of carbapenems increased from 0.048 DID in 2008 to 0.079 DID in 2012 (Yoon et al., 2015). The average consumption of carbapenems in the European hospital sector was 0.04 DID (country range: 0.01–0.13), with 11 countries showing significant increasing trends, but only two countries (Norway and Portugal) experienced significant declines between 2010 and 2019 (European Centre for Disease Prevention and Control, 2019). There are many reasons for these large differences, some of which are a reflection of cultural determinants, the prevalence of resistant Gram-negative bacteria, physicians’ medication habits, and the prevalence of infectious disease.

Our study found that both the level and trend of carbapenem consumption decreased after the promulgation of the expert consensus, but the differences were not statistically significant. This was slightly different from other studies. Zhang et al. found that the consumption of carbapenems increased significantly from 2011 to 2017 (Zhang et al., 2019). Similarly, a retrospective analysis of 586 hospitals from 28 provinces in China found that the largest relative increase was observed for carbapenems, 233.3% (from 0.03 DID in 2011 to 0.16 DID in 2018) (Wushouer et al., 2020). A study based on monthly surveillance data of antibiotic sales from 468 tertiary hospitals in 28 provinces of China found that the consumption of carbapenems was significantly increased from an average of 0.06 Defined Daily Doses per 1,000 inhabitants per day (DID)in 2011 to 0.15 DID in 2015 (Wushouer et al., 2017). This difference was related to the time, region, and research objects included in different studies. There was no significant change in the usage of carbapenems, partly due to the increase in extended-spectrum β-lactamase-producing Gram-negative bacteria (China Antimicrobial Surveillance Network, CHINET, 2020), rather than a lack of an effect of the expert consensus.

In addition, this study showed that the consumption of carbapenem in primary health institutes had increased, which need to be paid more attention to. As the last line of defence for the treatment of Gram-negative bacterial infections, the use of carbapenem should be strictly restricted. However, primary health institutes mainly provide medical services for patients with mild and moderate infections due to the scale of development and the ability of physicians, while patients with severe infections need to seek treatment in higher-level hospitals (Zhang, et al., 2020). Most of the existing studies on the analysis of carbapenem use are focused on secondary and higher medical institutions, while few studies on the use of carbapenem in primary institutions, with limited available data.

### The Effects on the Economic Burden of the Residents

This study found that the percentage of carbapenem expenditures to total antimicrobial expenditure decreased abruptly after the intervention, but the long-term trend was still upward, and which suggested that the long-term effects of the intervention were limited. It was found that total carbapenem expenditures in 2020 were slightly higher than those in 2019, but all were significantly lower than those in 2018. After the intervention, the increasing trend of total carbapenem expenditure slowed down. Time series analysis found that the intervention had a statistically significant impact on the long-term trend of total expenditure.

The vast majority of expenditures occurred in urban hospitals, including all tertiary hospitals, and some secondary hospitals in urban cities. This is consistent with the goal of hierarchical hospital management in China. Large hospitals are mainly responsible for the treatment of difficult and critical patients according to their scale and medical level, while carbapenems are known as the last line of defence for infection, so it is reasonable that carbapenems should be mainly used in urban hospitals (National Health Commission of the People’s Republic of China, 2020).

The expenditures for carbapenems during 2014–2016 were 139.4, 234.6 and 319.4 million US$, respectively, in tertiary hospitals in Shandong Province, showing the fastest-growing expenditure (Yin, et al., 2018). The expenditure on carbapenems in Shandong Province was significantly higher than the results of this study, which may be related to the differences in the study area and study time. The population of Shandong Province is approximately 2.5 times that of Shaanxi Province. It was found that the expert consensus had a positive impact on the economic burden of carbapenem usage by patients. After the issuance of the expert consensus, the level and trend of DDDc significantly decreased, and which means that this policy might promote the rational use of carbapenems. These findings indicate that the expert consensus played an important role in controlling carbapenem usage in public hospitals. However, the expenditure and consumption of carbapenems are still increasing, and scientific supervision is necessary.

This study indicated that the expert consensus has limited impact on the use and expenditure of carbapenems, which is not consistent with our previous assumptions as the administrative management is a very effective management measure in China. For example, the Ministry of Health launched a national campaign for rational antibiotic use in 2011. After the national campaign, the utilization of antibiotics has greatly been reduced (Xiao, 2018; Xiao et al., 2020). We believe that the main reason may be that this campaign did not require medical institutions to regularly feedback results and evaluate the feedback results. Therefore, we suggest that antimicrobial management in the future should be more scientific, professional and refined. The management of carbapenems should be continuously optimized, including setting specific indicators for different levels of medical institutions, and conducting continuous feedback and supervision. For medical institutions, it is time to establish a permanent task force professional team. Many studies (Clément et al., 2020; Sahil et al., 2020; Usman and Balamurugan, 2020) indicated that pharmacists played an irreplaceable role in promoting the rational use of antimicrobial through the feedback of the suitability of prescriptions and medical orders.

Moreover, realizing real-time prescription monitoring with the help of information technology also very important (Rittmann and Stevens, 2019), especially for restricting the use of carbapenems. It was found that the expenditure for carbapenems in the average value of baht per patient significantly decreased by 36.33% after using an antimicrobial restriction system (Wanla et al., 2017). Finally, as bacterial resistance is a constantly changing process, continuous training of tailored professional is needed.

### Limitations

This study has several limitations. First, data used in this study were not the actual use of carbapenem, but procurement records, so we did not calculate unreasonable utilization rates. And we did not analyse the evolution of bacterial resistance and its correlation with the use of carbapenem. It would be very helpful to conduct further research in prescription analysis and antimicrobial resistance monitoring, which can provide basis for clinical decision making. Second, we conducted this study in only one province; therefore, the results may not reflect the effect of the expert consensus throughout the nation. Third, as the centralized bidding procurement system did not cover all private healthcare institutions, the total carbapenem consumption may be underestimated. However, administrative measures are mainly aimed at public healthcare institutions, so it is of great significance to explore the impact of the introduction of the expert consensus on the change in carbapenem usage in public medical institutions.

## Conclusion

We revealed a short-term reduction in the usage and expenditures of carbapenems in public healthcare institutes in Shaanxi Province after the promulgation of the expert consensus that aimed to promote the rational use of carbapenems. However, the long-term effect was not optimal. To better promote the rational use of carbapenems, a series of strategies must be implemented. These strategies include government-oriented efforts to establish and optimize systems and infrastructure for the management of carbapenems use and monitoring of bacteria resistance, and continuous training of professional teams to ensure the sustainable management of carbapenems antimicrobial agents. Future studies should explore physician prescribing behaviour to fundamentally promote the appropriate use of carbapenems.

## Data Availability

The data analyzed in this study is subject to the following licenses/restrictions: The original datasets analyzed during the current study are not publicly available due to privacy restrictions but are available from the corresponding author on reasonable request. Requests to access these datasets should be directed to ykk10123@163.com.

## References

[B1] Centers for Disease Control and Prevention. (2019). Antibiotic Resistance Threats in the United States. [online]. Available at: https://www.cdc.gov/drugresistance/about.html .

[B2] China Antimicrobial Surveillance Network, CHINET. (2020). Bacterial Drug Resistance Monitoring Results of CHINET in 2020. [online]. Available at: http://www.chinets.com/Document .

[B3] Clément O. Nathanaël L. Marie A. Fernandez C. Dumartin C. Hindlet P. (2020). Pharmacists' Role in Antimicrobial Stewardship and Relationship with Antibiotic Consumption in Hospitals: an Observational Multicentre Study. J. Glob. Antimicrob. Resist. 20, 131–134. 10.1016/j.jgar.2019.07.009 31323427

[B4] European Centre for Disease Prevention and Control (ECDC). (2019). Antimicrobial Consumption-Annual Epidemiological Report for 2019. [online]. Available at: https://www.ecdc.europa.eu/sites/default/files/documents/Antimicrobial-consumption-in-the-EU-Annual-Epidemiological-Report-2019.pdf .

[B5] Hu Y. Ping Y. Li L. Xu H. Yan X. Dai H. (2016). A Retrospective Study of Risk Factors for Carbapenem-Resistant *Klebsiella pneumoniae* Acquisition Among ICU Patients. J. Infect. Dev. Ctries 10, 208–213. 10.3855/jidc.6697 27031451

[B6] Jiancong W. Mouqing Z. Fangfei L. (2020). Effect of Antibiotic Stewardship Programs on Reduction of Antimicrobial Resistance in China. Am. J. Infect. Control. 48 (2), 233–234. 31839275 10.1016/j.ajic.2019.11.013

[B7] Klein E. Y. Milkowska-Shibata M. Tseng K. K. Sharland M. Gandra S. Pulcini C. (2021). Assessment of WHO Antibiotic Consumption and Access Targets in 76 Countries, 2000-15: an Analysis of Pharmaceutical Sales Data. Lancet Infect. Dis. 21 (1), 107–115. 10.1016/S1473-3099(20)30332-7 32717205

[B8] Kuehn B. (2018). Antibiotic Resistance challenge. Jama 320 (18), 1851. 10.1001/jama.2018.16587 30422200

[B9] Liu P. Li X. Luo M. Xu X. Su K. Chen S. (2018). Risk Factors for Carbapenem-Resistant *Klebsiella pneumoniae* Infection: a Meta-Analysis. Microb. Drug Resist. 24, 190–198. 10.1089/mdr.2017.0061 28749714 PMC5873294

[B10] National Health and Family Planning Commission of China (2018a). Notification on Releasing 3 Technical Documents of Experts Options on Carbapenem Antibiotics. Beijing. [online]. No.822Available at: http://www.nhc.gov.cn/yzygj/s7659/201809/95f65ca473b44746b24590e94468b8ff.shtml .

[B11] National Health and Family Planning Commission of Shaanxi Province (2018b). Notification on Releasing 3 Technical Documents of Experts Options on Carbapenem Antibiotics. Xi'an. No.1049.

[B12] National Health Commission of the People's Republic of China. (2020). Evaluation Criteria for Grade III Hospitals (2020 Edition). [online]. Available at: http://www.nhc.gov.cn/yzygj/s7657/202012/b15e9333e72f426a81312d1d5c6c7250.shtml

[B13] O’Neill J. (2016). Tackling Drug-Resistant Infections Globally: Final Report and Recommendations. [online]. Available at: https://amr-review.org/sites/default/files/160518_Final%20paper_with%20cover.pdf .

[B14] Rittmann B. Stevens M. P. (2019). Clinical Decision Support Systems and Their Role in Antibiotic Stewardship: a Systematic Review. Curr. Infect. Dis. Rep. 21 (8), 29. 10.1007/s11908-019-0683-8 31342180

[B15] Sahil S. Michael M. Angela B. P. Sicard E. Fradette C. Zhao F. (2020). Pharmacist-driven Implementation of Fast Identification and Antimicrobial Susceptibility Testing Improves Outcomes for Patients with Gram-Negative Bacteremia and Candidemia. Antimicrob. Agents Chemother. 64 (9), e00578–20. 10.1128/AAC.00578-20 32601164 PMC7449197

[B16] Shaanxi Provincial Department of Health (2012). Guiding Opinions on Online Purchasing of Medicines in Urban Medical Institutions of Shaanxi Province. Xi'an. No.418.

[B17] The Statistical Yearbook in Shaanxi Province in 2019. (2019). The Statistical Yearbook in Shaanxi Province in 2019. [online]. Available at: http://tjj.shaanxi.gov.cn/ .

[B18] Usman A. Balamurugan T. (2020). Nationwide Survey of Pharmacists' Involvement in Antimicrobial Stewardship Programs in Nigerian Tertiary Hospitals[J]. J. Glob. Antimicrob. Resist. 21, 148–153. 10.1016/j.jgar.2019.10.007 31628999

[B19] Wagner A. K. Soumerai S. B. Zhang F. Ross-Degnan D. (2010). Segmented Regression Analysis of Interrupted Time Series Studies in Medication Use Research. J. Clin. Pharm. Ther. 27 (4), 299–309. 10.1046/j.1365-2710.2002.00430.x 12174032

[B20] Wanla W. Katip W. Supakul S. Apiwatnakorn P. Khamsarn S. (2017). Effects of an Antimicrobial Restriction System on Appropriate Carbapenem Use in a Hospital without Infectious Diseases Consultation. Int. J. Gen. Med. 10, 443–449. 10.2147/IJGM.S145133 29238212 PMC5713694

[B21] Watson R. (2017). European Commission Issues Advice on Use of Antimicrobials. BMJ 358, j3255. 10.1136/bmj.j3255 28676521

[B22] WHO (2013). Anatomical Therapeutic Chemical (ATC) Classification System:Guidelines for ATC Classification and DDD Assignment. Oslo: WHO Collaborating Centre for Drug Statisitcs Methodology.

[B23] WHO (2019). World Health Organization Model List of Essential Medicines, 21st List, 2019. Geneva: World Health Organization. https://aware.essentialmeds.org/groups.

[B24] Wushouer H. Tian Y. Guan X. D. Han S. Shi L. W. (2017). Trends and Patterns of Antibiotic Consumption in China's Tertiary Hospitals: Based on a 5 Year Surveillance with Sales Records, 2011-2015. PLoS One 12 (12), e0190314. 10.1371/journal.pone.0190314 29281716 PMC5744988

[B25] Wushouer H. Zhou Y. Zhang X. Fu M. Fan D. Shi L. (2020). Secular Trend Analysis of Antibiotic Utilisation in China's Hospitals 2011-2018, a Retrospective Analysis of Procurement Data. Antimicrob. Resist. Infect. Control. 9 (1), 53. 10.1186/s13756-020-00709-6 32295639 PMC7160954

[B26] Xiao Y. (2018). Antimicrobial Stewardship in China: Systems, Actions and Future Strategies. Clin. Infect. Dis. 67 (Suppl. l_2), S135–S141. 10.1093/cid/ciy641 30423041

[B27] Xiao Y. Shen P. Zheng B. Zhou K. Luo Q. Li L. (2020). Change in Antibiotic Use in Secondary and Tertiary Hospitals Nationwide after a National Antimicrobial Stewardship Campaign Was Launched in China, 2011-2016: An Observational Study. J. Infect. Dis. 221 (Suppl. 2), S148–S155. 10.1093/infdis/jiz556 32176788

[B28] Yin J. Wu C. Wei X. Sun Q. (2018). Antibiotic Expenditure by Public Healthcare Institutions in Shandong Province in China, 2012-2016. Front. Pharmacol. 9, 1396. 10.3389/fphar.2018.01396 30559665 PMC6287473

[B29] Yoon Y. K. Park G. C. An H. Chun B. C. Sohn J. W. Kim M. J. (20152015). Trends of Antibiotic Consumption in Korea According to National Reimbursement Data (2008-2012): A Population-Based Epidemiologic Study. Medicine (Baltimore) 94, e2100. 10.1097/MD.0000000000002100 PMC4652834 26579825

[B30] Zhang A. Nikoloski Z. Albala S. A. Yip W. Xu J. Mossialos E. (2020). Patient Choice of Health Care Providers in China: Primary Care Facilities versus Hospitals. Health Syst. Reform 6 (1), e1846844. 10.1080/23288604.2020.1846844 33314985

[B31] Zhang D. Hu S. Sun J. Zhang L. Dong H. Feng W. (2019). Antibiotic Consumption versus the Prevalence of Carbapenem-Resistant Gram-Negative Bacteria at a Tertiary Hospital in China from 2011 to 2017. J. Infect. Public Health 12 (2), 195–199. 10.1016/j.jiph.2018.10.003 30385238

[B32] Zhao H. Wei L. Li H. Zhang M. Cao B. Bian J. (2021). Appropriateness of Antibiotic Prescriptions in Ambulatory Care in China: a Nationwide Descriptive Database Study. Lancet Infect. Dis. 20, 1473–3099. 10.1016/S1473-3099(20)30596-X 33515511

