# Supplementary material for: Impact of the Expert Consensus on Carbapenem Consumption Trends and Patterns in Public Healthcare Institutes: An Interrupted Time Series Analysis, 2017–2020
Source: Front Pharmacol. 2022 Jan 13;12:739960. doi: 10.3389/fphar.2021.739960 (PMC8793738; doi:10.3389/fphar.2021.739960)
Supplement: Supplementary file 1 [file DataSheet1.docx]

Supplementary Table 1. Classification of antibiotics used in this study

| Antibiotic | Class | AWaRe category | Listed on  2019 AWaRe  classification  of antibiotics | Hierarchical management in China |
| --- | --- | --- | --- | --- |
| Tetracycline | Tetracyclines | Access | Yes | Non-Restricted |
| Doxycycline | Tetracyclines | Access | Yes | Non-Restricted |
| Oxytetracycline | Tetracyclines | Watch | Yes | Non-Restricted |
| Minocycline | Tetracyclines | Watch / Reserve | Yes | Restricted |
| Tigecycline | Glycylcycline | Reserve | Yes | Special |
| Chloramphenicol | Chloramphenicols | Access | Yes | Restricted |
| Amoxicillin | Broad-spectrum penicillins | Access | Yes | Non-Restricted |
| Ampicillin | Broad-spectrum penicillins | Access | Yes | Non-Restricted |
| Piperacillin | Broad-spectrum penicillins | Watch | Yes | Non-Restricted |
| Azlocillin | Broad-spectrum penicillins | Watch | Yes | Restricted |
| Mezlocillin | Broad-spectrum penicillins | Watch | Yes | Restricted |
| Sulbenicillin | Broad-spectrum penicillins | Watch | Yes | Restricted |
| Ticarcillin | Carbenicillins | Watch | Yes | Restricted |
| Penicillin G | Narrow-spectrum penicillins | Access | Yes | Non-Restricted |
| Penicillin V | Narrow-spectrum penicillins | Access | Yes | Non-Restricted |
| Oxacillin | Narrow-spectrum penicillins | Access | Yes | Non-Restricted |
| Cloxacillin | Narrow-spectrum penicillins | Access | Yes | Non-Restricted |
| Flucloxacillin | Narrow-spectrum penicillins | Access | Yes | Restricted |
| Amoxicillin/Clavulanic  Acid | Broad-spectrum penicillins | Access | Yes | Non-Restricted |
| Ampicillin/Sulbactam | Broad-spectrum penicillins | Access | Yes | Restricted |
| Piperacillin/Tazobactam | Broad-spectrum penicillins | Watch | Yes | Restricted |
| Cefalexin | Cephalosporins | Access | Yes | Non-Restricted |
| Cefazolin | Cephalosporins | Access | Yes | Non-Restricted |
| Cefradine | Cephalosporins | Access | Yes | Non-Restricted |
| Cefadroxil | Cephalosporins | Access | Yes | Non-Restricted |
| Cefathiamidine | Cephalosporins | Access | No | Restricted |
| Cefuroxime | Cephalosporins | Watch | Yes | Non-Restricted |
| Cefaclor | Cephalosporins | Watch | Yes | Non-Restricted |
| Cefprozil | Cephalosporins | Watch | Yes | Restricted |
| Cefotiam | Cephalosporins | Watch | Yes | Restricted |
| Ceftriaxone | Cephalosporins | Watch | Yes | Non-Restricted |
| Cefotaxime | Cephalosporins | Watch | Yes | Restricted |
| Cefixime | Cephalosporins | Watch | Yes | Restricted |
| Ceftazidime | Cephalosporins | Watch | Yes | Restricted |
| Cefdinir | Cephalosporins | Watch | Yes | Restricted |
| Ceftizoxime | Cephalosporins | Watch | Yes | Restricted |
| Cefpodoxime Proxetil | Cephalosporins | Watch | Yes | Restricted |
| Cefoperazone | Cephalosporins | Watch | Yes | Restricted |
| Cefepime | Cephalosporins | Watch | Yes | Special |
| Cefpirome | Cephalosporins | Watch | Yes | Special |
| Cefmetazole | Cephalosporins | Watch | Yes | Restricted |
| Cefoxitin | Cephalosporins | Watch | Yes | Restricted |
| Cefminox | Cephalosporins | Watch | Yes | Restricted |
| Latamoxef | Cephalosporins | Watch | Yes | Restricted |
| Faropenem | Cephalosporins | Reserve | Yes | Restricted (Oral)  Special (IV) |
| Aztreonam | Monobactams | Reserve | Yes | Special |
| Ertapenem | Carbapenems | Watch | Yes | Restricted |
| Meropenem | Carbapenems | Watch | Yes | Special |
| Imipenem/Cilastatin | Carbapenems | Watch | Yes | Special |
| Panipenem/Betamipron | Carbapenems | Watch | Yes | Special |
| Biapenem | Carbapenems | Watch | Yes | Special |
| Erythromycin | Macrolides | Watch | Yes | Non-Restricted |
| Azithromycin | Macrolides | Watch | Yes | Non-Restricted  (Oral)  Restricted(IV) |
| Acetylspiramycin | Macrolides | Watch | No | Non-Restricted |
| Roxithromycin | Macrolides | Watch | Yes | Non-Restricted |
| Clarithromycin | Macrolides | Watch | Yes | Non-Restricted |
| Dirithromycin | Macrolides | Watch | Yes | Restricted |
| Sulfadiazine/Trimethoprim | Trimethoprim | Access | No | Non-Restricted |
| Trimethoprim | Trimethoprim | Access | Yes | Non-Restricted |
| Clindamycin | Macrolide | Access | Yes | Non-Restricted |
| Lincomycin | Macrolide | Watch | Yes | Non-Restricted |
| Gentamicin | Aminoglycosides | Access | Yes | Non-Restricted |
| Amikacin | Aminoglycosides | Access | Yes | Non-Restricted |
| Streptomycin | Aminoglycosides | Watch | Yes | Non-Restricted |
| Neomycin | Aminoglycosides | Watch | Yes | Non-Restricted |
| Tobramycin | Aminoglycosides | Watch | Yes | Restricted |
| Etimicin | Aminoglycosides | Watch | No | Restricted |
| Netilmicin | Aminoglycosides | Watch | Yes | Restricted |
| Isepamicin | Aminoglycosides | Watch | Yes | Restricted |
| Spectinomycin | Other | Watch | Yes | Restricted |
| Ciprofloxacin | Fluoroquinolones | Watch | Yes | Non-Restricted |
| Norfloxacin | Fluoroquinolones | Watch | Yes | Non-Restricted |
| Levofloxacin | Fluoroquinolones | Watch | Yes | Non-Restricted |
| Ofloxacin | Fluoroquinolones | Watch | Yes | Non-Restricted |
| Moxifloxacin | Fluoroquinolones | Watch | Yes | Restricted |
| Antofloxacin | Fluoroquinolones | Watch | No | Restricted |
| Lomefloxacin | Fluoroquinolones | Watch | Yes | Special |
| Fleroxacin | Fluoroquinolones | Watch | Yes | Special |
| Gemifloxacin | Fluoroquinolones | Watch | Yes | Special |
| Colistin (oral) | Polymyxins | Reserve | Yes | Restricted |
| Colistin (injection) | Polymyxins | Reserve | Yes | Special |
| Polymyxin B | Polymyxins | Reserve | Yes | Special |
| Metronidazole | Other | Access | Yes | Non-Restricted |
| Fosfomycin | Phosphonics | Reserve (IV)/  Watch (Oral) | Yes | Non-Restricted |
| Rifampicin | Rifamycins | Watch | Yes | Restricted |
| Rifaximin | Rifamycins | Watch | Yes | Restricted |
| Rifamycin | Rifamycins | Watch | Yes | Restricted |
| Fusidic acid | Other | Watch | Yes | Special |
| Linezolid | Oxazolidinones | Reserve | Yes | Special |
| Daptomycin | Lipopeptides | Reserve | Yes | Special |

Supplementary Table 2. The impact of the implementation of the expert consensus on the use of carbapenems among urban hospitals in Shaanxi.

| Indicators | coefficient | t | p-value | 95% CI |
| --- | --- | --- | --- | --- |
| DID | | | | |
| β_0_ | 0.012421 | 8.68 | ＜0.001 | 0.0095376,0.0153044 |
| β_1_ | 0.0002442 | 2.37 | 0.022 | 0.0000364,0.000452 |
| β_2_ | -0.0014769 | -0.78 | 0.438 | -0.0052831,0.0023292 |
| β_3_ | -0.0001997 | -1.46 | 0.152 | -0.0004758,0.0000763 |
| Percentage of carbapenem expenditure to total antimicrobial expenditure | | | | |
| β_0_ | 0.0988373 | 16.26 | ＜0.001 | 0.0865836,0.111091 |
| β_1_ | -0.0000612 | -0.16 | 0.874 | -0.0008352,0.0007127 |
| β_2_ | -0.0187509 | -3.22 | 0.002 | -0.0304704,-0.0070314 |
| β_3_ | 0.0006444 | 1.36 | 0.180 | -0.000309,0.0015978 |
| Total expenditure | | | | |
| β_0_ | 7615099 | 8.35 | ＜0.001 | 5776339,9453859 |
| β_1_ | 217519.8 | 3.15 | 0.003 | 78137.62,356902 |
| β_2_ | -2582325 | -2.22 | 0.031 | -4922047,-242603.7 |
| β_3_ | -189127.8 | -2.20 | 0.033 | -362583.3,-15672.32 |
| DDDc | | | | |
| β_0_ | 533.1934 | 57.04 | ＜0.001 | 514.353,552.0338 |
| β_1_ | 2.934259 | 4.15 | ＜0.001 | 1.508529,4.35999 |
| β_2_ | -75.78942 | -3.43 | 0.001 | -120.3589,-31.21991 |
| β_3_ | -3.871496 | -2.84 | 0.007 | -6.618967,-1.124025 |

Supplementary Fig 1 The consumption of carbapenems among urban hospitals in Shaanxi Province, 2017–2020.

Supplementary Table 3. The impact of the implementation of the expert consensus on the use of carbapenems among county-level hospitals in Shaanxi.

| Indicators | coefficient | t | p-value | 95% CI |
| --- | --- | --- | --- | --- |
| DID | | | | |
| β_0_ | 0.0016618 | 6.82 | ＜0.001 | 0.0011711,0.0021525 |
| β_1_ | -7.67e-06 | -0.36 | 0.723 | -0.0000511,0.0000357 |
| β_2_ | 0.0006482 | 1.79 | 0.08 | -0.000081,0.0013774 |
| β_3_ | 2.29e-06 | 0.09 | 0.928 | -0.0000487,0.0000532 |
| Percentage of carbapenem expenditure to total antimicrobial expenditure | | | | |
| β_0_ | 0.0129534 | 4.64 | ＜0.001 | 0.0073306,0.0185763 |
| β_1_ | 0.0000578 | 0.21 | 0.833 | -0.000492,0.0006076 |
| β_2_ | -0.0012071 | -0.31 | 0.762 | -0.0091825,0.0067684 |
| β_3_ | 0.0000291 | 0.10 | 0.920 | -0.0005486,0.0006067 |
| Total expenditure | | | | |
| β_0_ | 745797.9 | 5.48 | ＜0.001 | 471589.4,1020006 |
| β_1_ | 1251.105 | 0.09 | 0.926 | -25679.67,28181.88 |
| β_2_ | 184107 | 0.91 | 0.369 | -224375.1,592589.1 |
| β_3_ | -2577.918 | -0.18 | 0.860 | -31759.79,26603.95 |
| DDDc | | | | |
| β_0_ | 394.5297 | 18.90 | ＜0.001 | 352.4497,436.6096 |
| β_1_ | 1.522284 | 0.96 | 0.344 | -1.681629,4.726197 |
| β_2_ | -37.5066 | -1.37 | 0.176 | -92.52672,17.51351 |
| β_3_ | -1.993564 | -0.99 | 0.328 | -6.053151,2.066022 |

Supplementary Fig 2 The consumption of carbapenems among county-level hospitals in Shaanxi Province, 2017–2020.

Supplementary Table 4. The impact of the implementation of the expert consensus on the use of carbapenems among primary health institutes in Shaanxi.

| Indicators | coefficient | t | p-value | 95% CI |
| --- | --- | --- | --- | --- |
| DID | | | | |
| β_0_ | 0.0000688 | 4.20 | ＜0.001 | 0.0000358,0.0001017 |
| β_1_ | -2.96e-06 | -2.37 | 0.022 | -5.47e-06,-4.47e-07 |
| β_2_ | 2.59e-06 | 0.12 | 0.904 | -0.0000405,0.0000457 |
| β_3_ | 4.00e-06 | 2.54 | 0.015 | 8.24e-07,7.18e-06 |
| Percentage of carbapenem expenditure to total antimicrobial expenditure | | | | |
| β_0_ | 0.0018 | 3.69 | 0.001 | 0.0008164,0.0027836 |
| β_1_ | -0.0000785 | -2.11 | 0.040 | -0.0001534,-3.60e-06 |
| β_2_ | -0.0000876 | -0.14 | 0.891 | -0.0013737,0.0011984 |
| β_3_ | 0.0001237 | 2.63 | 0.012 | 0.0000288,0.0002186 |
| Total expenditure | | | | |
| β_0_ | 0.0047653 | 3.70 | 0.001 | 0.0021707,0.0073599 |
| β_1_ | -0.0001975 | -2.01 | 0.050 | -0.000395,4.71e-08 |
| β_2_ | 0.0000688 | 0.04 | 0.968 | -0.0033236,0.0034612 |
| β_3_ | 0.0002673 | 2.15 | 0.037 | 0.000017,0.0005176 |
| DDDc | | | | |
| β_0_ | 424.7213 | 4.02 | ＜0.001 | 212.0141,637.4285 |
| β_1_ | -13.22981 | -1.62 | 0.112 | -29.64968,3.190053 |
| β_2_ | -10.8935 | -0.08 | 0.938 | -291.9552,270.1682 |
| β_3_ | 18.15579 | 1.77 | 0.084 | -2.511018,38.8226 |

Supplementary Fig 3 The consumption of carbapenems among primary health institutes in Shaanxi Province, 2017–2020.
